# Editorial: Brain-based mechanisms underlying the cognitive, sleep, and emotional benefits of aerobic exercise

**DOI:** 10.3389/fnhum.2026.2010856

**Published:** 2026-10-09

**Authors:** Keying Zhang, Chunmei Cao, Yih-Kuen Jan

**Affiliations:** 1 Department of Physical Education, Southeast University, Nanjing, China; 2 Division of Sports Science and Physical Education, Tsinghua University, Beijing, China; 3 Department of Kinesiology and Community Health, University of Illinois at Urbana-Champaign, Champaign, IL, United States

**Keywords:** aerobic exercise, brain plasticity, cognition, emotion, virtual reality

A central challenge in exercise neuroscience is to move beyond asking whether aerobic exercise benefits cognition, sleep, and emotional wellbeing toward understanding when these benefits emerge, for whom, at what dose, and through which neural processes. These outcomes may not respond uniformly to exercise or share the same neural correlates. The five articles in this Research Topic address these questions from complementary perspectives: three evidence syntheses, an EEG microstate study, and a multimodal MRI study. Together, they suggest that the links between exercise, brain function, and behavioral outcomes are unlikely to be explained by a single mechanism, but instead vary across outcomes, contexts, and timescales.

Across the outcome-focused articles, emotional effects are more consistently supported than cognitive or sleep outcomes. Wang et al. synthesized 19 randomized controlled trials in healthy adults and found that acute aerobic exercise increased positive mood and reduced negative mood, while longer-term exercise reduced negative mood. In contrast, pooled effects on cognition and sleep were not statistically significant. A second review of 14 randomized trials involving 982 university students found reductions in depressive symptoms, anxiety symptoms, and perceived stress, while dose-response analyses suggested that psychological benefits may vary with exercise prescription. The proposed dose ranges were appropriately presented as indicative rather than definitive thresholds (Yang et al).

These studies argue against treating aerobic exercise as a unitary exposure producing a unitary benefit. Some variation likely reflects differences in design, measurement, and intervention reporting, but reproducible differences across outcomes and populations may also contain biologically meaningful information. The challenge is to distinguish methodological noise from genuine variation in responsiveness. This is especially important for exercise dose, which combines intensity, duration, frequency, total intervention length, modality, and adherence.

The electrophysiological contributions extend the question from whether exercise changes brain activity to how such changes are expressed. Liu et al. synthesized 13 randomized controlled trials and found that exercise interventions were associated, on average, with greater EEG alpha-band power. However, heterogeneity was substantial and the prediction interval crossed the null. Because the review included exercise modalities beyond aerobic exercise, its findings are better interpreted as broad evidence of exercise-related electrophysiological modulation than as an aerobic-specific neural signature. The sensitivity of alpha power to recording state and analytic choices further cautions against treating a single spectral measure as a context-free biomarker.

Yao et al. examined a different dimension of EEG organization in adolescents with depressive symptoms and healthy peers who completed traditional and virtual-reality-enhanced aerobic cycling. Both conditions improved mood, while VR exercise was accompanied by changes in EEG microstate dynamics, including reduced microstate C occurrence, prolonged microstate D duration, and altered state transitions. Selected microstate measures were also associated with post-exercise mood. By focusing on the occurrence, duration, and transitions of electrophysiological states, this study shifts attention from signal magnitude toward temporal organization. These findings remain candidate neural correlates, but they raise a testable hypothesis: immediate psychological responses to exercise may be reflected partly in the way large-scale neural states reorganize over time.

Zhang et al. combined structural MRI, resting-state fMRI, and graded exercise testing in 32 men spanning high-level endurance athletes, moderately trained runners, and active controls. Aerobic-capacity indicators were associated with gray-matter volume, fALFF, and degree centrality across distributed cortical and cerebellar regions, with a more selective pattern after multiple-comparison correction. The contribution is not evidence for a defined training trajectory, but a broader view of aerobic fitness as a distributed brain-physiology phenotype. Descriptively different associations across training levels were less stable in sensitivity analyses and should remain hypothesis-generating.

Read alongside the EEG studies, these findings raise a multiscale question: how are rapid neural responses to a single bout of exercise related, if at all, to the brain characteristics observed across different levels of fitness and training experience?

Taken together, the five articles reveal both progress and an uneven evidence landscape. Emotional outcomes are the most consistently supported behavioral domain in this collection, whereas cognition is less uniform and sleep remains only weakly connected to direct mechanistic evidence. The neural studies point toward distributed and dynamic correlates rather than a single region, frequency band, or biomarker, yet methodological heterogeneity, limited longitudinal evidence, modest sample sizes, and the correlational nature of several brain-behavior relationships continue to constrain mechanistic interpretation.

The next step is not simply to accumulate more evidence that exercise is beneficial, but to connect currently separated levels of explanation. Future studies should integrate multidimensional exercise dose, physiological responses, behavioral outcomes, and multimodal brain measures within randomized and longitudinal designs, while distinguishing acute neural responses from adaptations that emerge with repeated training. One emerging experimental opportunity is the integration of virtual reality with aerobic exercise. Beyond engagement, VR may allow contextual and sensory features to be manipulated while key exercise parameters are matched, helping test whether similar physiological demands produce different neural and psychological responses under different experiential conditions. A useful working framework is therefore to examine exercise as a multiscale process in which dose and context shape physiological state, acute neural dynamics, repeated adaptation, and ultimately behavioral outcome. The key question is whether transient exercise-related neural responses can be linked prospectively to durable adaptation and to individual differences in cognitive, sleep, and emotional benefit.

